# Pirin: a potential novel therapeutic target for castration‐resistant prostate cancer regulated by *miR‐455‐5p*

**DOI:** 10.1002/1878-0261.12405

**Published:** 2018-12-21

**Authors:** Takayuki Arai, Satoko Kojima, Yasutaka Yamada, Sho Sugawara, Mayuko Kato, Kazuto Yamazaki, Yukio Naya, Tomohiko Ichikawa, Naohiko Seki

**Affiliations:** ^1^ Department of Functional Genomics Chiba University Graduate School of Medicine Japan; ^2^ Department of Urology Chiba University Graduate School of Medicine Japan; ^3^ Department of Urology Teikyo University Chiba Medical Center Ichihara Japan; ^4^ Department of Pathology Teikyo University Chiba Medical Center Ichihara Japan

**Keywords:** bisamide, castration‐resistant prostate cancer, *miR‐455‐5p*, pirin

## Abstract

Androgen deprivation therapy is frequently used to treat prostate cancer (PCa), but resistance can occur, a condition known as castration‐resistant prostate cancer (CRPC). Thus, novel approaches for identification of CRPC are important for designing effective PCa treatments. Analysis of microRNA (miRNA) expression signatures by RNA sequencing showed that both passenger and guide strands of the *miR‐455*‐duplex (*miR‐455‐5p* and *miR‐455‐3p*, respectively) acted as antitumor miRNAs in PCa cells. The involvement of miRNA passenger strands in cancer pathogenesis is a novel concept for miRNA functionality. Based on a large patient cohort in The Cancer Genome Atlas, expression of eight *miR‐455‐5p/‐3p* target genes (*PIR*:* P *=* *0.0137, *LRP8*:* P *=* *0.0495, *IGFBP3*:* P *=* *0.0172, *DMBX1*:* P *=* *0.0175*, CCDC64*:* P *=* *0.0446, *TUBB1*:* P *=* *0.0149, *KIF21B*:* P *=* *0.0336, and *NFAM1*:* P *=* *0.0013) was significantly associated with poor prognosis of PCa patients. Here, we focused on *PIR* (pirin), a highly conserved member of the cupin superfamily. *PIR* expression was directly regulated by *miR‐455‐5p*, and PIR overexpression was detected in hormone‐sensitive prostate cancer (HSPC) surgical specimens and CRPC autopsy specimens. Loss‐of‐function assays using siRNA or an inhibitor (bisamide) showed that downregulation of *PIR* expression blocked cancer cell migration and invasion. Moreover, the *miR‐455‐5p*/*PIR* axis contributed to cancer cell aggressiveness. These results suggest that *PIR* might be a promising diagnostic marker for HSPC and CRPC. Furthermore, CRPC treatment strategies targeting *PIR* may be possible in the future. Identification of antitumor miRNAs, including miRNA passenger strands, may contribute to the development of new diagnostic markers and therapeutic strategies for CRPC.

**Abbreviations**
ADT: androgen deprivation therapy
AR: androgen receptor
CRPC: castration‐resistant prostate cancer
DFS: disease‐free survival
HSF1: heat shock transcriptional factor 1
HSPC: hormone‐sensitive prostate cancer
miRNA: microRNA
PCa: prostate cancer
PIR: pirin
TCGA: The Cancer Genome Atlas

## 1 Introduction

Due to the expansion of prostate‐specific antigen (PSA) screening, prostate cancer (PCa) is now the most frequently diagnosed cancer among men in developed countries. In 2017, PCa was the third leading cause of cancer‐related death among men (approximately 320 000 individuals) in the United States (Siegel *et al*., 2017). Activation of androgen signaling through the androgen receptor (AR) is essential for development, proliferation, and survival of PCa cells. Therefore, AR is considered to be the most relevant therapeutic target for both hormone‐sensitive prostate cancer (HSPC) and advanced PCa (Crawford *et al*., 2015; Crona and Whang, 2017). Most HSPC patients initially respond to androgen deprivation therapy (ADT), but eventually PCa cells acquire resistance to ADT and gain increased proliferative and metastatic potential, a condition referred to as castration‐resistant prostate cancer (CRPC). Recently approved agents such as second generation AR‐targeted agents, bone‐targeted agents and chemotherapeutic agents increase survival rates of CRPC patients, but do not provide a cure (Crawford *et al*., 2015; Crona *et al*., 2015). Thus, increasing the likelihood of survival for CRPC patients is an important consideration for successful PCa treatments.

Many studies showed that microRNAs (miRNAs), a type of small noncoding RNA, are associated with cancer pathogenesis (Esquela‐Kerscher and Slack, 2006; Iorio and Croce, 2009; Nelson and Weiss, 2008; Wiemer, 2007). A single miRNA species can regulate many protein‐coding or noncoding RNA transcripts in physiological and pathological conditions (Friedman *et al*., 2009). Therefore, novel RNA networks in cancer cells can be searched beginning with aberrantly expressed miRNAs.

Based on HSPC and CRPC miRNA signatures, we sequentially identified antitumor miRNAs and the oncogenic targets they control, including *FSCN1*,* GOLM1*,* PNP*,* WWP1*, and *Ecm29* (Fuse *et al*., 2011; Goto *et al*., 2015, 2016; Kojima *et al*., 2012, 2014). Our recent RNA sequencing analyses of CRPC miRNA signatures revealed that expression of both strands of pre‐*miR‐145* (*miR‐145‐5p* and *miR‐145‐3p*, the passenger and guide strand, respectively) was significantly reduced in HSPC and CRPC clinical specimens and that *miR‐145‐5p/‐3p* acted as an antitumor miRNA in PCa cells (Goto *et al*., 2017). Interestingly, ectopic expression of *miR‐145‐3p* markedly blocked cancer cell aggressiveness through direct regulation of several oncogenic genes, including *MELK*,* NCAPG*,* BUB1*, and *CDK1* (Goto *et al*., 2017). Recent studies of miRNA biogenesis showed that some miRNA passenger strands, which were previously thought to be degraded and not functioning, also had important roles in human cells, including cancer cells (Marzi *et al*., 2016; McCall *et al*., 2017).

In this study, our aim was to identify novel therapeutic targets and prognostic markers for CRPC. We demonstrated that both *miR‐455*‐duplex strands (*miR‐455‐5p*, the passenger strand; and *miR‐455‐3p*, the guide strand) possess antitumor functions. We also found some molecular targets of *miR‐455‐5p/‐3p* to reveal new characteristics of PCa pathogenesis. Expression of eight genes (*PIR*,* LRP8*,* IGFBP3*,* DMBX1*,* CCDC64*,* TUBB1*,* KIF21B*, and *NFAM1*) was regulated by *miR‐455‐5p/‐3p*, and high expression of these genes was significantly predictive of survival in PCa patients. Moreover, we validated the functional significance of *PIR* as a promising therapeutic target for CRPC.

## 2 Materials and methods

### 2.1 Collection of clinical prostate specimens and cell lines

Clinical specimens were provided by the Teikyo University Chiba Medical Center between 2013 and 2017. Table S1 lists the clinical characteristics of these patients. The research protocol was approved by the Teikyo University Institutional Review Committee. The experiments were undertaken with the understanding and written consent of each subject, and the study methodologies conformed to the standards set by the Declaration of Helsinki.

We used human PCa cell lines (PC3, DU145, and C4‐2) obtained from the Cell Resource Center for Biomedical Research, Institute of Development, Aging and Cancer Tohoku University (Sendai, Japan), and the American Type Culture Collection (Manassas, VA, USA). The cells were maintained as described in our previous reports (Arai *et al*., 2018b; Goto *et al*., 2017; Kurozumi *et al*., 2016; Okato *et al*., 2017a; Yamada *et al*., 2018b).

### 2.2 Quantitative real‐time reverse transcription polymerase chain reaction

Expression levels of *miR‐455‐5p* and *miR‐455‐3p* normalized to expression of *RNU4*8 were analyzed by TaqMan quantitative real‐time reverse transcription polymerase chain reaction (qRT‐PCR). *PIR* expression levels were normalized to *GAPDH* or *GUSB*. The procedure for qRT‐PCR quantification was described in our previous reports (Arai *et al*., 2017, 2018b; Goto *et al*., 2017; Kurozumi *et al*., 2016; Okato *et al*., 2017a; Yamada *et al*., 2018b). Details of the reagents used are shown in Table S2.

### 2.3 Transfection with mature miRNA, small‐interfering RNA, or plasmid vectors

For experiments involving mature miRNAs, small‐interfering RNAs (siRNAs), and plasmid vectors, we used pre‐miR miRNA precursors (*hsa‐miR‐455‐5p* and *hsa‐miR‐455‐3p*), Stealth Select RNAi siRNAs (si‐*PIR‐*1 and si‐*PIR‐*2), and negative control miRNA/siRNA as well as a *PIR* plasmid vector designed by Kazusa DNA Research (Product ID: FHC03682; Kisarazu, Japan). miRNAs or siRNAs were introduced into cells at a concentration of 10 nm by reverse transfection, and a vector plasmid was introduced into cells by forward transfection. The procedures were as previously reported (Arai *et al*., 2017, 2018b; Goto *et al*., 2017; Kurozumi *et al*., 2016; Okato *et al*., 2017a; Yamada *et al*., 2018b), and the reagent details are given in Table S2.

### 2.4 Small‐molecule PIR inhibitor bisamide for *in vitro* studies

Bisamide, which was previously reported to be a small‐molecule PIR inhibitor, was used to inhibit PIR in *in vitro* assays (CCT251236; MedChem Express Monmouth Junction, NJ, USA; Cat No. 1693731‐40‐6; Cheeseman *et al*., 2017). Bisamide was dissolved in DMSO, and the final DMSO concentration in experiments involving bisamide was ≤ 0.1%.

### 2.5 Cell proliferation, migration, and invasion assays

To investigate the functional roles of miRNAs or siRNAs in PCa cells, cell proliferation (XTT assay), migration (wound‐healing assay), and invasion (Matrigel invasion assay) assays were carried out as previously described (Arai *et al*., 2017, 2018b; Goto *et al*., 2017; Kurozumi *et al*., 2016; Okato *et al*., 2017a; Yamada *et al*., 2018b).

### 2.6 Validation of miRNAs incorporated into the RNA‐induced silencing complex

To explore whether both exogenous strands of pre‐*miR‐455* (*miR‐455‐5p* and *miR‐455‐3p*) were incorporated into the RNA‐induced silencing complex (RISC), we performed Ago2 immunoprecipitation assays using a miRNA isolation kit for human Ago2 (Wako, Osaka, Japan). The procedure was described previously (Goto *et al*., 2017; Okato *et al*., 2017a). Quantification of miRNAs bound to Ago2 was achieved by qRT‐PCR, and normalization was performed relative to the expression of *miR‐26a* that is unaffected by transfection with *miR‐455‐5p/‐3p*. Details of the reagents used are shown in Table S2.

### 2.7 Strategy for exploring genes regulated by *miR‐455‐5*p, *miR‐455‐3*p si‐*PIR*, and PIR inhibitor in PCa cells

We combined *in silico* database analyses and comprehensive gene expression analyses using an oligo microarray (Agilent Technologies, Tokyo, Japan; Human Ge 60K) to focus on target gene candidates as previously described (Arai *et al*., 2017, 2018a,b; Kurozumi *et al*., 2016; Okato *et al*., 2017a; Yamada *et al*., 2018b). For *in silico* analyses, we used the TargetScanHuman 7.1 database (June, 2016 release, http://www.targetscan.org/vert_71). The microarray data were deposited into the GEO database (https://www.ncbi.nlm.nih.gov/geo/).

### 2.8 Western blotting

Western blotting was carried out as previously described with anti‐PIR antibodies (diluted to 1 : 400) and anti‐glyceraldehyde 3‐phosphate dehydrogenase (GAPDH) antibodies (diluted to 1 : 10 000) as an internal loading control (Arai *et al*., 2017, 2018b; Kurozumi *et al*., 2016; Okato *et al*., 2017a; Yamada *et al*., 2018b). Antibody details are described in Table S2.

### 2.9 Plasmid construction and dual‐luciferase reporter assays

A partial wild‐type sequence of the *PIR* 3′‐untranslated region (UTR) or a sequence having a mutation in the *miR‐455‐5p* target site was inserted into the psiCHECK‐2 vector (C8021; Promega, Madison, WI, USA). The assay procedure was reported previously (Arai *et al*., 2017, 2018b; Kurozumi *et al*., 2016; Okato *et al*., 2017a; Yamada *et al*., 2018b).

### 2.10 Immunohistochemistry

Tissue specimens were incubated overnight at 4 °C with anti‐PIR antibodies (diluted to 1 : 80). Tissue microarrays were obtained from provitro (Cat. no. 4012209, Berlin, Germany). Details of the tissue microarray are given in http://www.provitro.com/fileadmin/provitro-data/TMA/4012209.pdf. The reagents used are described in Table S2, and the procedure was described previously (Arai *et al*., 2017, 2018a,b; Goto *et al*., 2017; Kurozumi *et al*., 2016; Okato *et al*., 2017a; Yamada *et al*., 2018b). IHC score was evaluated by weighted intensity. The intensity of staining was graded from 0 to 3 as follows: 0, negative staining; 1, mild staining; 2, moderate staining; and 3, intense staining. The procedure was performed as described previously (Okato *et al*., 2016).

### 2.11 The Cancer Genome Atlas database analyses of PCa

To investigate the clinical significance of miRNAs and genes in PCa patients, we utilized the The Cancer Genome Atlas (TCGA) database. Gene expression data and clinical information for PCa patients were analyzed using cBioPortal (http://www.cbioportal.org/; Gao *et al*., 2013). The data were downloaded on August 29, 2017. The expression of each gene was divided into two groups, high and low, and the period preceding postoperative recurrence (disease‐free survival) was compared and examined.

### 2.12 Statistical analysis

The relationship between two groups was analyzed using the Mann–Whitney *U*‐test. The relationship of three or more variables was analyzed using Bonferroni‐adjusted Mann–Whitney *U*‐tests. The correlation between two groups was evaluated by Spearman's rank test. Survival analyses by Kaplan–Meier method and log‐rank test were performed using jmp software (version 13; SAS Institute Inc., Cary, NC, USA). For all other analyses, expert statview (version 5; SAS Institute, Inc.) was used.

## 3 Results

### 3.1 Expression levels of both pre‐*miR‐455* strands in PCa specimens and cell lines

**Figure 1:**
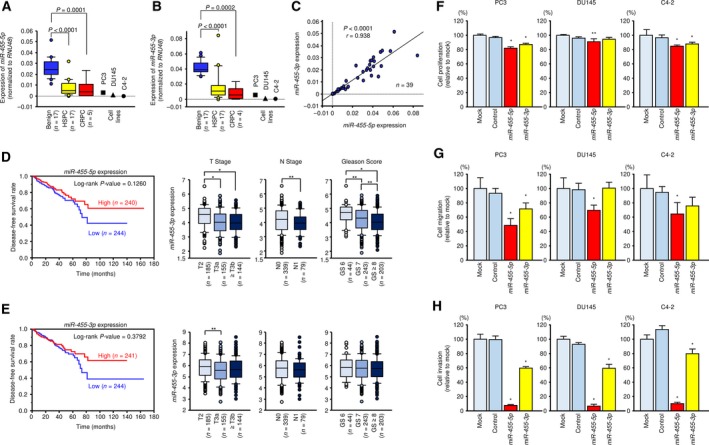
Expression of *miR‐455‐5p/‐3p* in clinical PCa specimens and functional analysis of *miR‐455‐5p/‐3p* in PCa cell lines. Expression levels of (A) *miR‐455‐5p* and (B) *miR‐455‐3p* in PCa clinical specimens and cell lines determined using qRT‐PCR. *RNU48* was used as an internal control. *P*‐values were calculated using Bonferroni‐adjusted Mann–Whitney *U*‐test. (C) Correlations between the relative expression levels of *miR‐455‐5p* and *miR‐455‐3p* analyzed by using Spearman's rank test. (D) Kaplan–Meier patient survival curves for DFS rates based on *miR‐455‐5p* expression (left) and relationships between *miR‐455‐5p* expression and T stage, N stage, and Gleason score (right) in PCa patients from the TCGA database. (E) Kaplan–Meier patient survival curves for DFS rates based on *miR‐455‐3p* expression (left) and relationships between expression of *miR‐455‐3p* and T stage, N stage, and Gleason score (right). *P <* *0.0001 and **P <* *0.05. *P*‐values were calculated using Mann–Whitney *U*‐test or Bonferroni‐adjusted Mann–Whitney *U*‐test. (F–H) Cell proliferation, migration, and invasion assays in cells transfected with *miR‐455‐5p/‐3p*. Error bars are represented as mean ± SD (*n* = 5, *n* = 8, and *n* = 8, respectively). *P <* *0.0001 and **P <* *0.05, relative to both mock and control. *P*‐values were calculated using Bonferroni‐adjusted Mann–Whitney *U*‐test.

In the human genome, pre‐*miR‐455* is located on chromosome 9q32 and the mature sequences of *miR‐455‐5p* and *miR‐455‐3p* are 5′‐UAUGUGCCUUUGGACUACAUCG‐3′ and 5′‐GCAGUCCAUGGGCAUAUACAC‐3′, respectively (Fig. S1). *miR‐455‐5p* is the passenger strand (minor strand), and *miR‐455‐3p* is the guide strand (major strand). We validated *miR‐455‐5p* and *miR‐455‐3p* expression levels in clinical prostate specimens [benign prostate tissues: *n* = 17; HSPC: *n* = 17; and CRPC: *n* = 5; Table S1] and PCa cell lines (PC3, DU145, and C4‐2). qRT‐PCR indicated that *miR‐455‐5p* and *miR‐455‐3p* expression was markedly downregulated in HSPC and CRPC tissues compared with benign prostate tissues (*miR‐455‐5p*:* P *<* *0.0001 and *P *=* *0.0001; *miR‐455‐3p*:* P *<* *0.0001 and *P *=* *0.0002; Fig. 1A,B). PCa cell lines also had very low expression levels of both *miR‐455‐5p* and *miR‐455‐3p* (Fig. 1A,B). Moreover, *miR‐455‐5p* and *miR‐455‐3p* expression was positively correlated in prostate tissues (*r* = 0.938, *P *<* *0.0001; Fig. 1C). In general, the expression levels of miRNA passenger strands in clinical specimens are quite low, although here the expression levels of *miR‐455‐5p* and *miR‐455‐3p* were comparable. This result suggests that both the passenger strand and the guide strand may play important functional roles in PCa.

### 3.2 Binding of both pre‐*miR‐455* strands to Ago2

To confirm that both *miR‐455‐5p* and *miR‐455‐3p* function by incorporating into the RISC, we performed immunoprecipitation with antibodies targeting Ago2, a central player for miRNA incorporation into the RISC (Fig. S2A). qRT‐PCR showed that the amount of *miR‐455‐5p* bound to Ago2 was significantly higher in *miR‐455‐5p* transfectants compared to mock‐transfected cells and cells transfected with miR‐control and *miR‐455‐3p* (*P *<* *0.0001; Fig. S2B). Similarly, the amount of *miR‐455‐3p* bound to Ago2 was markedly higher in *miR‐455‐3p* transfectants than that of mock‐, miR‐control‐, and *miR‐455‐5p‐*transfected cells (*P *<* *0.0001; Fig. S2B).

### 3.3 The clinical significance of *miR‐455‐5p/‐3p* in PCa patients and functional effects of restoring *miR‐455‐5p/‐3p* in PCa cells

To investigate the clinical significance of *miR‐455‐5p* and *miR‐455‐3p* in PCa patients, we carried out several analyses using the TCGA database. There was no significance in disease‐free survival (DFS) rate in the expression difference of *miR‐455‐5p*, but the prognosis tended to be poor in PCa patients with lower levels of *miR‐455‐5p* expression (Fig. 1D). Additionally, analyses of T stage, N stage, and Gleason score showed that *miR‐455‐5p* expression was markedly decreased in advanced PCa cases or cases with high malignancy (Fig. 1D). On the other hand, no clinically significant differences were seen for *miR‐455‐3p* expression in terms of survival or stage (Fig. 1E).

To confirm the functional roles of *miR‐455‐5p* and *miR‐455‐3p*, we performed ectopic expression assays and evaluated the cell proliferation, migration, and invasion activity in PCa cells (PC3, DU145, and C4‐2). Our ectopic expression assays showed that *miR‐455‐5p* inhibited cancer cell migration and invasive abilities in all PCa cells (Fig. 1G,H). In contrast to *miR‐455‐5p*, the inhibitory effects of the migration and invasive abilities of *miR‐455‐3p* were inferior in all PCa cells (Fig. 1G,H). In addition, both miRNAs had only slight suppression effects on cell proliferation in PCa cells (Fig. 1F).

These results suggested that *miR‐455‐5p* is clinically important in PCa and acts as an antitumor miRNA to suppress cancer cell migration and invasive abilities *in vitro*.

### 3.4 Search for putative oncogenes regulated by *miR‐455‐5p/‐3p* in PCa cells

**Figure 2:**
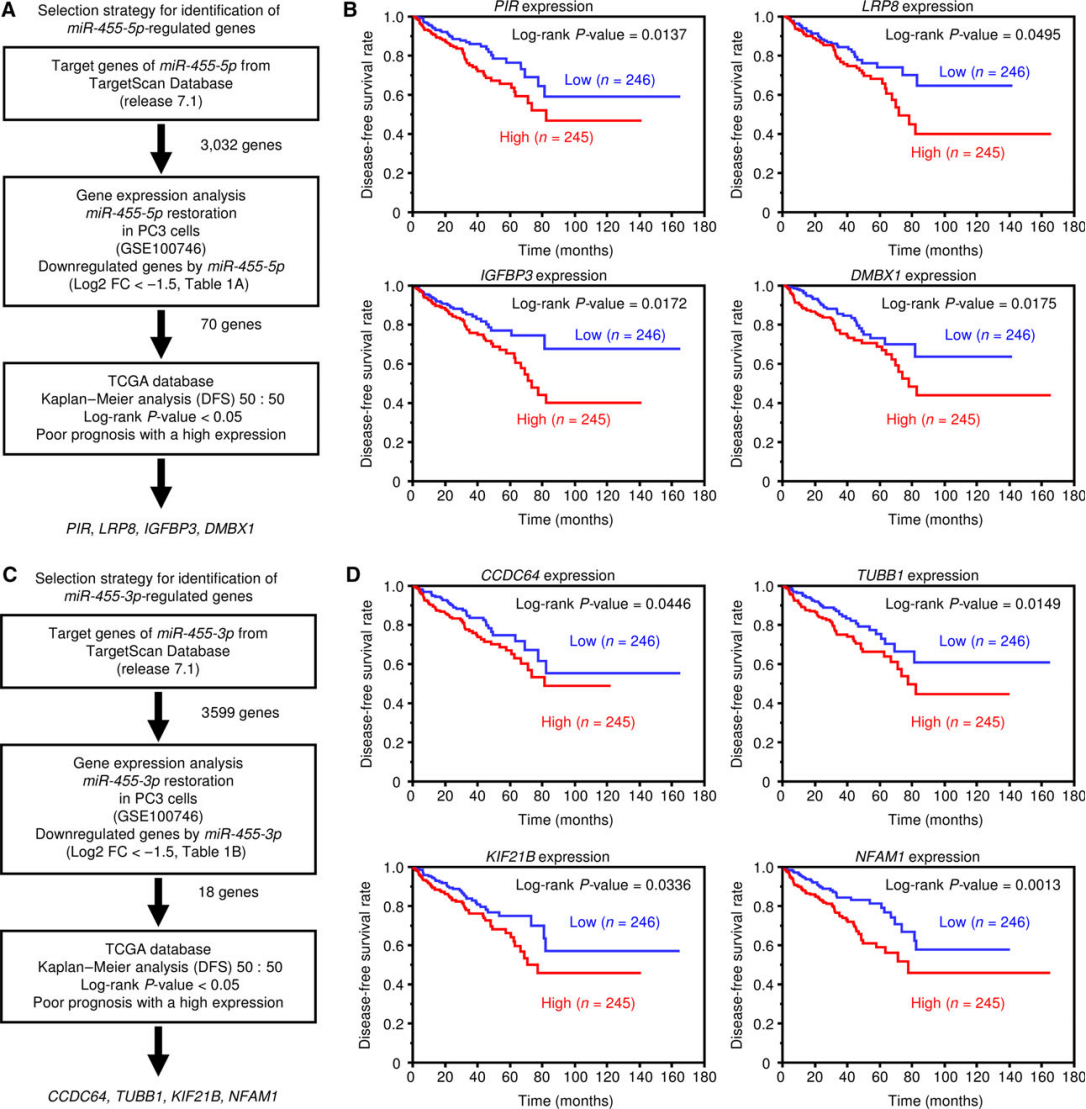
Identification of *miR‐455‐5p/‐3p* target genes and relationship between putative target genes and DFS rates. (A) Flowchart of the strategy used to identify *miR‐455‐5p* target genes. (B) Kaplan–Meier patient survival curves for DFS rates based on *PIR, LRP8, IGFBP3*, and *DMBX1* expression in PCa patients from the TCGA database. (C) Flowchart of the strategy to identify *miR‐455‐3p* target genes. (D) Kaplan–Meier patient survival curves for DFS rates based on *CDC64, TUBB1, KIF21B*, and *NFAM1* expression.

**Figure 3:**
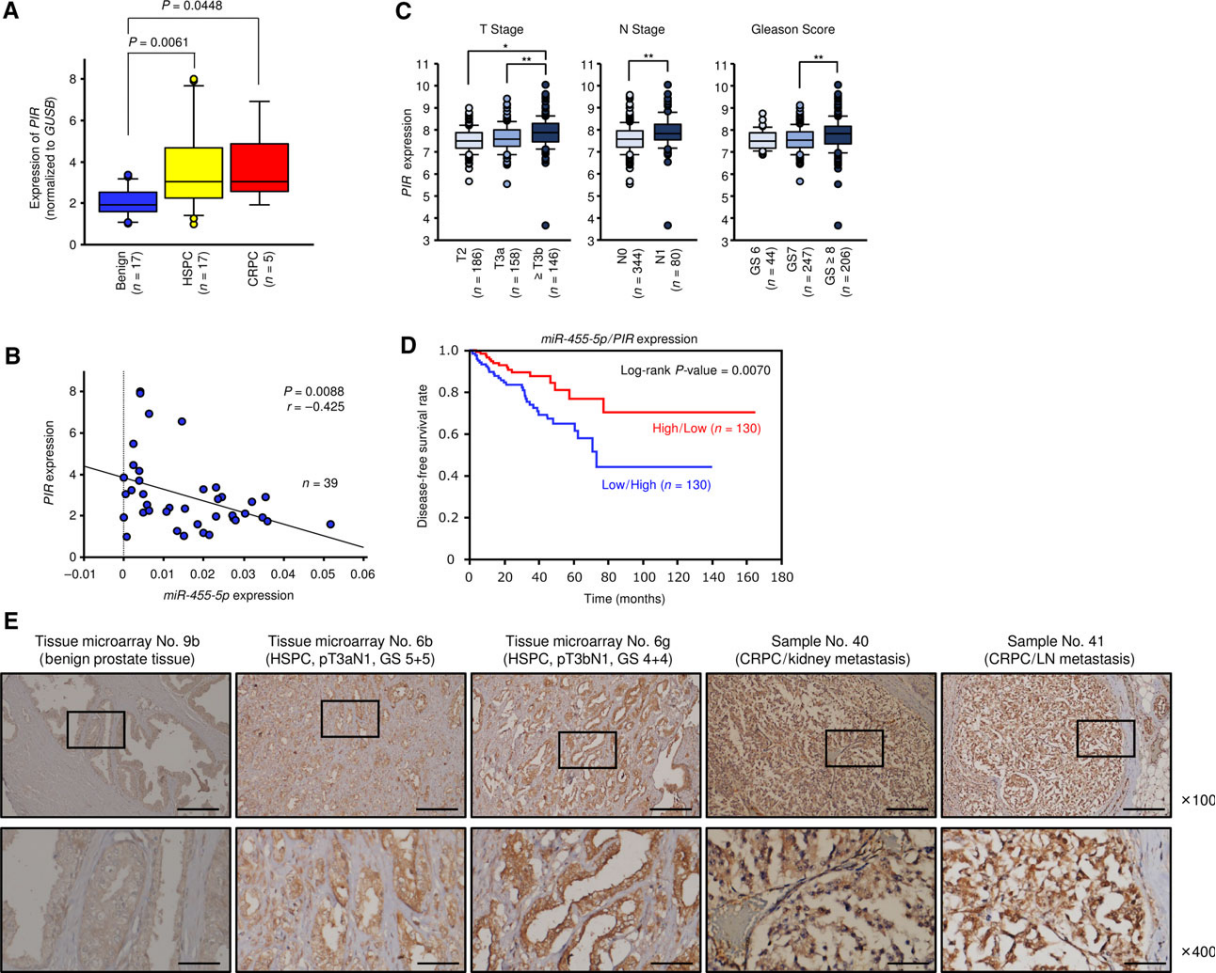
Expression of PIR in clinical PCa specimens and relationship between *PIR* and clinicopathological factors. (A) Expression levels of *PIR* in PCa clinical specimens and cell lines. *GUSB* was used as an internal control. *P*‐values were calculated using Bonferroni‐adjusted Mann–Whitney *U*‐test. (B) Negative correlation between *miR‐455‐5p* and *PIR* expression analyzed by using Spearman's rank test. (C) Relationships between *PIR* expression and T stage, N stage, and Gleason score in PCa patients from the TCGA database. *P <* *0.0001, **P <* *0.001. *P*‐values were calculated using Mann–Whitney *U*‐test or Bonferroni‐adjusted Mann–Whitney *U*‐test. (D) Kaplan–Meier patient survival curves for DFS rates by a combination of *miR‐455‐5p* and *PIR* expression (*miR‐455‐5p* high/*PIR* low versus *miR‐455‐5p* low/*PIR* high). (E) Immunochemical staining of PIR in prostate specimens. Scale bars of ×100 and ×400 represent 200 and 50 μm, respectively.

To identify oncogenes regulated by *miR‐455‐5p*, we searched the TargetScanHuman 7.1 database and compiled a list of 3032 candidate genes having sites in the 3′‐UTR that are bound by *miR‐455‐5p*. From these candidate genes, we selected 70 genes that also showed downregulated expression (fold change log_2_^ ^< −1.5) in PCa cells transfected with *miR‐455‐5p* (GEO accession number: GSE100746; Table 1A). Then, we analyzed the patient's DFS in the TCGA database and selected genes significantly associated with poor prognosis when expressed at high level. The flowchart of the search is shown in Fig. 2A. These analyses identified four genes: *PIR*,* LRP8*,* IGFBP3*, and *DMBX1* (Fig. 2B). We also used the same method for *miR‐455‐3p* and identified four candidate oncogenes: *CCDC64*,* TUBB1*,* KIF21*B, and *NFAM1* (Fig. 2C,D and Table 1B). The results of clinicopathological analyses in PCa patients (T stage, N stage, and Gleason score) for the expression of these eight genes are shown in Fig. 3C and Fig. S3.

**Table 1:** Putative target genes regulated by (A) *miR‐455‐5p* and (B) *miR‐455‐3p* in PCa cells

(A)						
Entrez gene ID	Gene symbol	Gene name	Location	No. conserved sites	No. poorly conserved sites	PC3 *miR‐455‐5p* transfectant (Log_2_ ratio)
5738	*PTGFRN*	Prostaglandin F2 receptor inhibitor	1p13.1	1	0	−2.78
137075	*CLDN23*	Claudin 23	8p23.1	0	1	−2.73
11031	*RAB31*	RAB31, member RAS oncogene family	18p11.22	0	3	−2.63
66008	*TRAK2*	Trafficking protein, kinesin binding 2	2q33.1	1	1	−2.63
7084	*TK2*	Thymidine kinase 2, mitochondrial	16q21	0	2	−2.56
10959	*TMED2*	Transmembrane emp24 domain trafficking protein 2	12q24.31	1	1	−2.45
3964	*LGALS8*	Lectin, galactoside‐binding, soluble, 8	1q43	0	1	−2.44
4201	*MEA1*	Male‐enhanced antigen 1	6p21.1	0	1	−2.41
9563	*H6PD*	Hexose‐6‐phosphate dehydrogenase (glucose 1‐dehydrogenase)	1p36.22	0	1	−2.38
141	*ADPRH*	ADP‐ribosylarginine hydrolase	3q13.33	0	2	−2.37
2820	*GPD2*	Glycerol‐3‐phosphate dehydrogenase 2 (mitochondrial)	2q24.1	0	2	−2.33
8544	*PIR*	Pirin (iron‐binding nuclear protein)	Xp22.2	0	1	−2.29
29943	*PADI1*	Peptidyl arginine deiminase, type I	1p36.13	0	1	−2.29
7804	*LRP8*	Low‐density lipoprotein receptor‐related protein 8, apolipoprotein e receptor	1p32.3	0	2	−2.28
314	*AOC2*	Amine oxidase, copper containing 2 (retina‐specific)	17q21.31	0	1	−2.25
4038	*LRP4*	Low‐density lipoprotein receptor‐related protein 4	11p11.2	0	1	−2.17
64506	*CPEB1*	Cytoplasmic polyadenylation element binding protein 1	15q25.2	1	0	−2.15
8000	*PSCA*	Prostate stem cell antigen	8q24.3	0	1	−2.14
8895	*CPNE3*	Copine III	8q21.3	0	1	−2.11
8228	*PNPLA4*	Patatin‐like phospholipase domain containing 4	Xp22.31	0	1	−2.08
127343	*DMBX1*	Diencephalon/mesencephalon homeobox 1	1p33	0	2	−2.05
54749	*EPDR1*	Ependymin related 1	7p14.1	0	2	−1.98
3992	*FADS1*	Fatty acid desaturase 1	11q12.2	0	2	−1.97
659	*BMPR2*	Bone morphogenetic protein receptor, type II (serine/threonine kinase)	2q33.2	0	2	−1.96
1047	*CLGN*	Calmegin	4q31.1	0	1	−1.94
100128927	*ZBTB42*	Zinc finger and BTB domain containing 42	14q32.33	0	2	−1.94
10584	*COLEC10*	Collectin subfamily member 10 (C‐type lectin)	8q24.12	0	2	−1.90
143903	*LAYN*	Layilin	11q23.1	1	1	−1.87
22873	*DZIP1*	DAZ interacting zinc finger protein 1	13q32.1	0	1	−1.84
79718	*TBL1XR1*	Transducin (beta)‐like 1 X‐linked receptor 1	3q26.32	1	0	−1.84
56180	*MOSPD1*	Motile sperm domain containing 1	Xq26.3	0	1	−1.84
3486	*IGFBP3*	Insulin‐like growth factor binding protein 3	7p12.3	0	1	−1.82
84327	*ZBED3*	Zinc finger, BED‐type containing 3	5q13.3	0	2	−1.80
203447	*NRK*	Nik‐related kinase	Xq22.3	0	1	−1.78
1238	*ACKR2*	Atypical chemokine receptor 2	3p22.1	0	1	−1.77
9194	*SLC16A7*	Solute carrier family 16 (monocarboxylate transporter), member 7	12q14.1	0	3	−1.76
128989	*TANGO2*	Transport and golgi organization 2 homolog (Drosophila)	22q11.21	0	1	−1.74
9870	*AREL1*	Apoptosis‐resistant E3 ubiquitin protein ligase 1	14q24.3	0	1	−1.72
5500	*PPP1CB*	Protein phosphatase 1, catalytic subunit, beta isozyme	2p23.2	0	1	−1.71
6444	*SGCD*	Sarcoglycan, delta (35 kDa dystrophin‐associated glycoprotein)	5q33.3	0	1	−1.70
4942	*OAT*	Ornithine aminotransferase	10q26.13	0	1	−1.66
116071	*BATF2*	Basic leucine zipper transcription factor, ATF‐like 2	11q13.1	0	1	−1.65
286077	*FAM83H*	Family with sequence similarity 83, member H	8q24.3	0	1	−1.64
414149	*ACBD7*	Acyl‐CoA binding domain containing 7	10p13	0	2	−1.64
100132386	*KRTAP4‐9*	Keratin associated protein 4‐9	17q21.2	0	2	−1.63
55163	*PNPO*	Pyridoxamine 5′‐phosphate oxidase	17q21.32	0	1	−1.63
23414	*ZFPM2*	Zinc finger protein, FOG family member 2	8q22.3	1	0	−1.63
26049	*FAM169A*	Family with sequence similarity 169, member A	5q13.3	0	2	−1.63
83394	*PITPNM3*	PITPNM family member 3	17p13.2	0	1	−1.63
132228	*LSMEM2*	Leucine‐rich single‐pass membrane protein 2	3p21.31	0	1	−1.61
8428	*STK24*	Serine/threonine kinase 24	13q32.2	1	0	−1.60
4352	*MPL*	Myeloproliferative leukemia virus oncogene	1p34.2	0	1	−1.60
1901	*S1PR1*	Sphingosine‐1‐phosphate receptor 1	1p21.2	1	1	−1.60
9258	*MFHAS1*	Malignant fibrous histiocytoma amplified sequence 1	8p23.1	1	0	−1.59
9779	*TBC1D5*	TBC1 domain family, member 5	3p24.3	0	1	−1.58
57722	*IGDCC4*	Immunoglobulin superfamily, DCC subclass, member 4	15q22.31	0	1	−1.57
144110	*TMEM86A*	Transmembrane protein 86A	11p15.1	1	1	−1.57
2782	*GNB1*	Guanine nucleotide binding protein (G protein), beta polypeptide 1	1p36.33	0	1	−1.56
255027	*MPV17L*	MPV17 mitochondrial membrane protein‐like	16p13.11	0	1	−1.56
81031	*SLC2A10*	Solute carrier family 2 (facilitated glucose transporter), member 10	20q13.12	0	1	−1.55
9214	*FAIM3*	Fas apoptotic inhibitory molecule 3	1q32.1	0	1	−1.55
153339	*TMEM167A*	Transmembrane protein 167A	5q14.2	1	0	−1.54
8498	*RANBP3*	RAN binding protein 3	19p13.3	1	0	−1.54
11337	*GABARAP*	GABA(A) receptor‐associated protein	17p13.1	0	1	−1.54
83445	*GSG1*	Germ cell associated 1	12p13.1	0	2	−1.53
142940	*TRUB1*	TruB pseudouridine (psi) synthase family member 1	10q25.3	0	1	−1.53
10423	*CDIPT*	CDP‐diacylglycerol‐inositol 3‐phosphatidyltransferase	16p11.2	0	1	−1.53
4155	*MBP*	Myelin basic protein	18q23	0	1	−1.52
374900	*ZNF568*	Zinc finger protein 568	19q13.12	0	1	−1.52
9658	*ZNF516*	Zinc finger protein 516	18q23	1	1	−1.51

(B)						
Entrez gene ID	Gene symbol	Gene name	Location	No. conserved sites	No. poorly conserved sites	PC3 *miR‐455‐3p* transfectant (Log_2_ ratio)
9887	*SMG7*	SMG7 nonsense‐mediated mRNA decay factor	1q25.3	0	1	−2.24
81033	*KCNH6*	Potassium voltage‐gated channel, subfamily H (eag‐related), member 6	17q23.3	0	1	−2.08
8778	*SIGLEC5*	Sialic acid binding Ig‐like lectin 5	19q13.41	0	1	−2.02
92558	*CCDC64*	Coiled‐coil domain containing 64	12q24.23	0	1	−1.88
23201	*FAM168A*	Family with sequence similarity 168, member A	11q13.4	0	2	−1.86
50615	*IL21R*	Interleukin‐21 receptor	16p12.1	0	1	−1.81
56479	*KCNQ5*	Potassium voltage‐gated channel, KQT‐like subfamily, member 5	6q13	0	3	−1.81
7099	*TLR4*	Toll‐like receptor 4	9q33.1	0	2	−1.77
85441	*HELZ2*	Helicase with zinc finger 2, transcriptional coactivator	20q13.33	0	1	−1.76
339327	*ZNF546*	Zinc finger protein 546	19q13.2	0	1	−1.76
79663	*HSPBAP1*	HSPB (heat shock 27 kDa) associated protein 1	3q21.1	1	0	−1.75
81027	*TUBB1*	Tubulin, beta 1 class VI	20q13.32	0	1	−1.71
23046	*KIF21B*	Kinesin family member 21B	1q32.1	0	2	−1.71
1236	*CCR7*	Chemokine (C‐C motif) receptor 7	17q21.2	0	1	−1.66
1002	*CDH4*	Cadherin 4, type 1, R‐cadherin (retinal)	20q13.33	0	1	−1.61
1238	*ACKR2*	Atypical chemokine receptor 2	3p22.1	0	1	−1.59
150372	*NFAM1*	NFAT activating protein with ITAM motif 1	22q13.2	0	3	−1.53
283078	*MKX*	Mohawk homeobox	10p12.1	0	1	−1.51

### 3.5 Prognostic application of genes regulated by miRNAs

We then attempted to determine whether useful prognostic evaluations could be made by examining the expression levels of each gene targeted by *miR‐455‐5p* or *miR‐455‐3p*. To evaluate gene expression, we created heatmaps by cluster analyses using R2: Genomics Analysis and Visualization Platform (https://r2.amc.nl/; Fig. S4A,C). For each of the four candidate genes for *miR‐455‐5p* and *miR‐455‐3p* regulation, we determined the averages of the *Z*‐scores and analyzed the DFS based on the resulting value (*Z*‐score < 0 versus ≥ 0). The candidate genes regulated by *miR‐455‐5p* and *miR‐455‐3p* showed significant differences in expression (*P *<* *0.0001 and *P *<* *0.0001, respectively; Fig. S4B,D). In particular, the Kaplan–Meier curve for DFS generated from a combination of the four candidate target genes for *miR‐455‐5p* showed a great impact.

Here, we focused on genes that were regulated by *miR‐455‐5p* and are thought to be important in controlling PCa progression. Among the four final candidate oncogenes, *PIR* showed the highest *miR‐455‐5p*‐mediated downregulation of expression and had the most significant effect on prognosis according to a log‐rank test.

### 3.6 *PIR* expression was directly regulated by *miR‐455‐5p* in PCa cells

To confirm the control of *PIR* expression by *miR‐455‐5p*, we performed qRT‐PCR and western blotting. Both *PIR* mRNA and protein expression levels were significantly reduced by *miR‐455‐5p* transfection compared to those of mock‐ or miR‐control‐transfected cells (Fig. S5A,B).

According to the TargetScan database, *miR‐455‐5p* binds to position 167–173 in the 3′‐UTR of *PIR*. To validate the direct binding of *miR‐455‐5p* to *PIR* mRNA, we performed luciferase reporter assays, which showed that the luminescence intensity was markedly decreased by cotransfection with *miR‐455‐5p* and a vector carrying wild‐type *PIR* 3′‐UTR. On the other hand, cells cotransfected with a vector carrying a mutated *miR‐455‐5p* target site showed no change in luminescence intensity (Fig. S5C).

### 3.7 *PIR* expression in PCa clinical specimens

We evaluated *PIR* expression in PCa clinical specimens (benign prostate tissues: *n* = 17; HSPC tissues: *n* = 17; and CRPC tissues: *n* = 5; Table S1). *PIR* expression was significantly upregulated in HSPC as compared to normal tissues and tended to be increased in CRPC tissues (*P *=* *0.0061 and *P *=* *0.0448, respectively; Fig. 3A). A Spearman's rank test showed a significant inverse correlation between *PIR* and *miR‐455‐5p* expression (*P *=* *0.0088, *r* = −0.425; Fig. 3B).

Immunohistochemistry analysis of PIR protein expression in PCa clinical specimens (tissue microarray and Table S1) indicated strong expression of the PIR protein in the cytoplasm of PCa cells (Fig. 3E). According to evaluation using tissue microarray, the expression score for PIR protein was significantly higher in PCa tissues than in noncancerous tissues (Fig. S6A). The patient backgrounds and IHC scores of PIR are summarized in Table S3, and representative immunostaining pictures are shown in Fig. S6B–D.

### 3.8 Clinical significance of *PIR* in PCa

As mentioned above, high expression of *PIR* was associated with poor prognosis in DFS (Fig. 2B). According to the TCGA database, *PIR* expression was enhanced in cases with advanced T stage, advanced N stage, and high Gleason score (Fig. 3C). Patients having low *miR‐455‐5p*/high *PIR* expression had significantly shorter DFS than the high‐*miR‐455‐5p*/low‐*PIR* group (Fig. 3D), suggesting that by combination of these two factors, it would be possible to predict the prognosis of PCa patients more effectively than to evaluate by *PIR* alone.

### 3.9 Effects of *PIR* silencing in PCa cell lines

**Figure 4:**
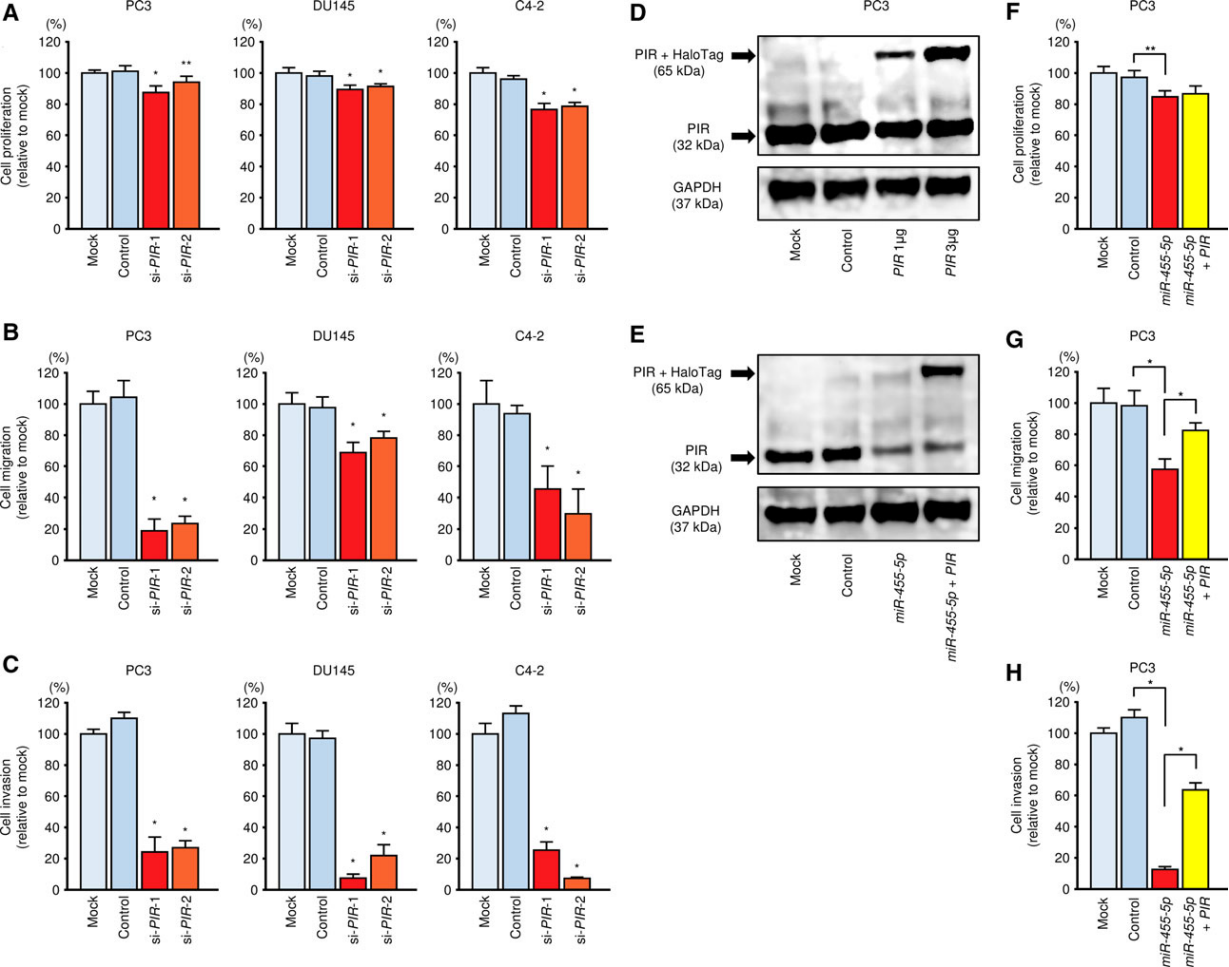
Functional analysis of *PIR* in PCa cells. (A–C) Knockdown assay with si‐*PIR*. Cell proliferation, migration, and invasion assays following transfection with si‐*PIR*‐1 and si‐*PIR‐*2. Error bars are represented as mean ± SD (*n* = 5, *n* = 8, and *n* = 8, respectively). *P <* *0.0001 and **P <* *0.001, relative to both mock and control. *P*‐values were calculated using Bonferroni‐adjusted Mann–Whitney *U*‐test. (D) PIR protein expression was evaluated by western blot analysis of PC3 cells 48 h after forward transfection with the *PIR* vector. GAPDH was used as a loading control. (E) PIR protein expression was evaluated by western blot analysis of PC3 cells 72 h after reverse transfection with *miR‐455‐5p* and 48 h after forward transfection with the *PIR* vector. (F–H) Rescue experiments with *miR‐455‐5p* and *PIR* vector. Cell proliferation, migration, and invasion assays following transfection with *miR‐455‐5p* and *PIR* vector. Error bars are represented as mean ± SD (*n* = 5, *n* = 8, and *n* = 8, respectively). *P <* *0.0001, **P <* *0.001. *P*‐values were calculated using Bonferroni‐adjusted Mann–Whitney *U*‐test.

We examined the effects of *PIR* knockdown in PC3, DU145, and C4‐2 cells using two types of si‐*PIR*: si‐*PIR‐*1 and si‐*PIR*‐2. Both siRNAs effectively downregulated *PIR* mRNA and PIR protein expression (Fig. S7A,B). Additionally, functional assays showed antitumor effects, particularly reduced cell migration and invasion, upon *PIR* knockdown (Fig. 4A–C).

### 3.10 Effects of *PIR/miR‐455‐5p* cotransfection of PC3 cells

We performed *PIR* rescue experiments by cotransfection with *PIR* and *miR‐455‐5p* in PC3 cells. Western blot was used to confirm the restoration of PIR protein levels (Fig. 4D,E). Although proliferation rates were not noticeably affected by *PIR* rescue (Fig. 4F), the migration and invasion capacities of PC3 cells were recovered by cotransfection with *PIR* and *miR‐455‐5p* (Fig. 4G,H), suggesting that the *miR‐455‐5p/PIR* axis may play an important role in PCa progression.

### 3.11 Antitumor effects using a small‐molecule PIR inhibitor in PC3 cells

We examined the tumor‐suppressive effects *in vitro* using the recently described small‐molecule PIR inhibitor bisamide (CCT251236; Cheeseman *et al*., 2017). In PC3, DU145, and C4‐2 cells using bisamide, the expression of *PIR* at the mRNA level was decreased in a concentration‐dependent manner (Fig. S8). We evaluated cell proliferation, migration (wound‐healing), and invasion activities at different bisamide concentrations ranging from 1 to 100 nm in PC3, DU145, and C4‐2 cells. In the wound‐healing assay, the cells were plated at high confluency to reduce the influence of cell proliferation suppression and bisamide was administered after scratching.

**Figure 5:**
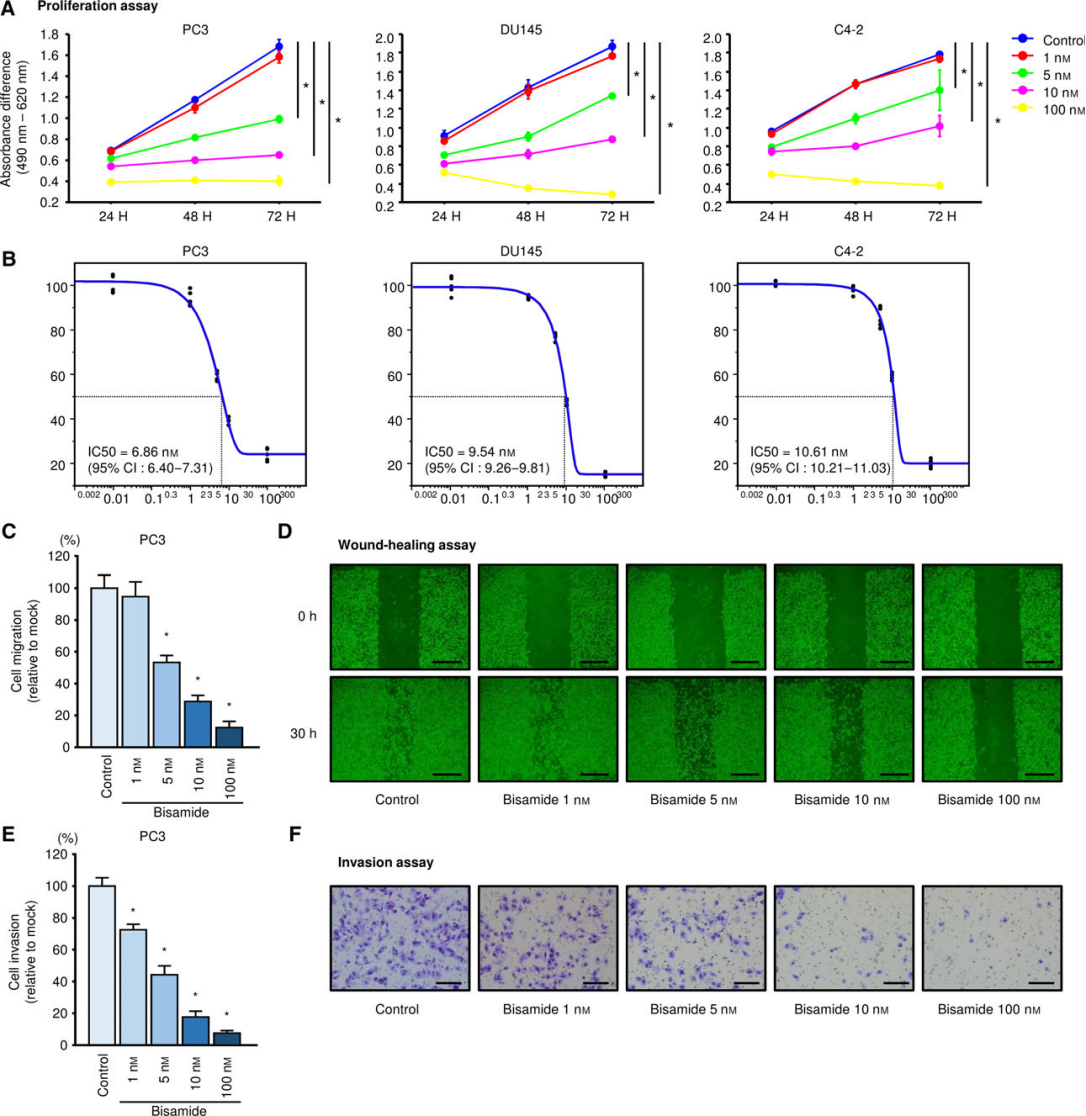
Effects of the small‐molecule PIR inhibitor bisamide (CCT251236) on PCa proliferation, migration, and invasion. (A) Proliferation curves over time according to the results of XTT assays following bisamide treatment in PC3, DU145, and C4‐2 were generated using the absorbance difference between 490 and 620 nm. Error bars are represented as mean ± SD (*n* = 5). *P <* *0.0001, relative to control at 72 h. *P*‐values were calculated using Bonferroni‐adjusted Mann–Whitney *U*‐test. (B) Dose‐dependent curves of bisamide on cell proliferation at 72 h in PC3, DU145, and C4‐2. The IC50 was calculated using jmp software. (C) Cell migration assays using bisamide in PC3. Error bars are represented as mean ± SD (*n* = 8). *P <* *0.0001, relative to control. *P*‐values were calculated using Bonferroni‐adjusted Mann–Whitney *U*‐test. (D) Phase micrographs of wound‐healing assays using bisamide in PC3. Scale bars represent 500 μm. (E) Cell invasion assays using bisamide in PC3. Error bars are represented as mean ± SD (*n* = 8). *P <* *0.0001, relative to control. *P*‐values were calculated using Bonferroni‐adjusted Mann–Whitney *U*‐test. (F) Phase micrographs of invasion assays using bisamide in PC3. Scale bars represent 200 μm.

First, the result of the XTT assay over time is shown in Fig. 5A. In all cell lines, bisamide suppressed cell proliferation in a concentration‐dependent manner, and its ability was almost lost at concentrations above 100 nm. A dose‐dependent curve in each cell line at 72 h is shown in Fig. 5B. Furthermore, as a result of evaluating migration and invasive abilities using PC3 cells, they were similarly suppressed by concentration dependence of bisamide (migration; Fig. 5C,D; and invasion; Fig. 5E,F). The aggressiveness of cancer cells disappeared under 100 nm bisamide or more. The dramatic effects on migration and invasion were thought to be due to the failure of the cells to proliferate in the presence of high concentrations of bisamide.

### 3.12 Search for genes downstream of *PIR*

We next used microarray analyses with si‐*PIR* or bisamide (GEO accession number: GSE115801 and GSE115800, respectively) to identify downstream genes affected by *PIR* in PCa cells (Fig. S9A; Tables [Link], [Link]–S6). Interestingly, *ACTL8* was detected by si‐*PIR* and bisamide mediated downstream gene in PCa cells (Tables [Link], [Link]–S6). *ACTL8* belongs to the cancer testis antigen (CTA) family (Yao *et al*., 2014). Although biological roles of CTAs are hardly elucidated, it is known as a multifunctional protein group having a specific expression pattern in various types of cancer cells (Gordeeva, 2018). At this time, functional significance of *ACTL8* for PCa cells is unknown. In the future, detailed functional analysis of *ACTL8* in PCa cells is necessary.

Given that bisamide is an inhibitor of heat shock transcriptional factor 1 (HSF1) stress pathways (Cheeseman *et al*., 2017), we investigated its effect on HSF1‐mediated genes, particularly for those genes registered in the Reactome pathway ‘Cellular response to heat stress’ (https://reactome.org/). The expression of most HSF1‐mediated genes was indeed downregulated in the presence of bisamide (Fig. S9B and Table S7).

## 4 Discussion

Specific features of miRNAs allow a single miRNA to regulate many different RNA transcripts under various physiological or pathological conditions. Based on this broad regulation, novel RNA networks can be identified from relevant miRNAs in cancer cells. To determine which miRNAs are dysregulated in cancer cells, we used RNA sequencing technologies to identify miRNA expression signatures in drug‐resistant clinical specimens and autopsy specimens. Analyses of our previously identified miRNA signatures highlighted novel RNA networks that are controlled by novel miRNAs in several cancers (Goto *et al*., 2017; Koshizuka *et al*., 2017b; Mizuno *et al*., 2017).

In earlier studies, we made the unexpected discovery that some miRNA passenger strands have antitumor activity, for example, *miR‐145‐3p*,* miR‐150‐3p*,* miR‐149‐3p*, and *miR‐99a‐3p*, in several cancer cells (Arai *et al*., 2018b; Goto *et al*., 2017; Koshizuka *et al*., 2017b; Okato *et al*., 2017b). The traditional view of miRNA function was that only one strand of the miRNA‐duplex is incorporated into the RISC to become the active strand (guide strand). In contrast, the other strand, the passenger strand or miRNA*, was thought to be degraded and to have no function (Chendrimada *et al*., 2005; Hutvagner and Zamore, 2002; Matranga *et al*., 2005). Our recent studies showed that both strands of the miRNA‐duplex, for example, *miR‐145*,* miR‐223*,* miR‐150*, and *miR‐199*, have antitumor functions and their guide and passenger strands control several oncogenes independently or jointly (Goto *et al*., 2017; Koshizuka *et al*., 2017a,b; Sugawara *et al*., 2018).

Here, we focused on the *miR‐455*‐duplex (*miR‐455‐5p*, the passenger strand; and *miR‐455‐3p*, the guide strand) based on our original CRPC signature (Goto *et al*., 2017). The functional assays in this study showed that both *miR‐455* strands had tumor‐suppressive functions in PCa cells. In particular, *miR‐455‐5p*, the passenger strand, strongly suppressed the migratory and invasive ability of PCa cells. Previous studies showed that downregulation of *miR‐455‐3p* (the guide strand) expression is frequently seen in several cancers, for example, colon cancer, non‐small‐cell lung cancer, and esophageal squamous cell carcinoma, and that ectopic expression of *miR‐455‐3p* inhibited the metastatic activity of cancer cells (Gao *et al*., 2018; Yang *et al*., 2017; Zheng *et al*., 2016). In PCa cells, *miR‐455‐3p* inhibits cancer cell proliferation by targeting the transcription factor *eIF4E* (Zhao *et al*., 2017). Interestingly, *miR‐455‐5p* had an opposite function depending on cancer type. For example, *miR‐455‐5p* expression is upregulated in NSCLC and could enhance cancer proliferation and metastasis by suppressing SOCO3 expression (Wang *et al*., 2017). In oral squamous cell carcinoma, *miR‐455‐5p* expression was promoted by the regulation of the TGF‐β‐dependent pathway and contributed to cancer tumorigenesis by downregulating *UBE2B* expression (Cheng *et al*., 2016). However, *miR‐455‐5p* expression in gastric cancer was lower, and thus, in cancer *miR‐455‐5p* functions as a tumor suppressor through regulation of *RAB18* (Liu *et al*., 2016). Our recent study demonstrated that expression of both strands of the *miR‐455*‐duplex was downregulated in renal cell carcinoma tissues and that ectopic expression of both miRNAs inhibited cancer cell migration and invasive abilities (Yamada *et al*., 2018a). The best part of miRNAs study is to identify responsible target genes and pathways controlled by miRNAs in cells.

In this study, exploration of the RNA network controlled by antitumor *miR‐455‐5p* and *miR‐455‐3p* expands our understanding of the novel molecular pathogenesis of HSPC and CRPC. Through our search strategies, we identified *PIR*,* LRP8*,* IGFBP3*, and *DMBX1* as genes controlled by *miR‐455‐5p*, and *CCDC64*,* TUBB1*,* KIF21B*, and *NFAM1* as genes controlled by *miR‐455‐3p*. According to analyses based on the TCGA database, these genes may have cancer‐promoting activity because their high expression correlates with poor prognosis of PCa patients. Interestingly, by combining the expression of these genes, survival curves for DFS showed more impressive results. In particular, the combination of the four target genes of *miR‐455‐5p* had a great impact. This may lead to the establishment of a strong prognostic biomarker by analyzing molecules originating from miRNAs. In this study, we focused on *PIR*, which was most strongly regulated by *miR‐455‐5p*, and proceeded the analyses.

*PIR* (pirin) is a member of the cupin superfamily and is a highly conserved gene among mammals, plants, fungi, and prokaryotes (Wendler *et al*., 1997). Previous reports suggested that *PIR* contributes to malignant transformation of cancer cells by acting as a transcriptional cofactor that interacts with the Bcl3–NF‐қB complex in several cancers (Adeniran and Hamelberg, 2017; Komai *et al*., 2015; Qiao *et al*., 2014). Additionally, a previous study showed that knockdown of *PIR* or treatment with the small molecule markedly suppressed migration of melanoma cells (Miyazaki *et al*., 2010). Furthermore, *PIR* inhibited the expression of E‐cadherin and induced the epithelial to mesenchymal transition phenotype in HeLa cells (Komai *et al*., 2015). According to the TCGA database, high expressions of *PIR* were significantly associated with short overall survival in glioblastoma, low‐grade glioma, and melanoma patients (Fig. S10). In PCa, *PIR* was reported to enhance cancer cell proliferation by negatively regulating apoptosis induced by EAF2/U19 (Qiao *et al*., 2014). However, the functions of *PIR*, including the mechanisms by which *PIR* controls metastasis in PCa, remain unclear. Our present data showed that siRNA‐mediated *PIR* knockdown significantly inhibited cancer cell aggressiveness. Surprisingly, transfection with *miR‐455‐5p* or si‐*PIR* produced a similar phenotype in cells lines that did (e.g., C4‐2) and did not express AR (e.g., PC3, DU145). Additionally, rescue experiments showed that the migration and invasive abilities of PCa cells that were suppressed by transfection with *miR‐455‐5p* were remarkably recovered by supplementing with *PIR*, suggesting that the *miR‐455‐5p/PIR* axis plays an important role in cancer progression, especially metastasis and invasion of PCa, through AR‐independent pathways. These results also suggest that *PIR* could be a target molecule for treatment of various cancers, including CRPC. Therefore, strategies to inhibit *PIR* expression are needed.

The chemical probe bisamide (CCT251236) bound PIR in a functional screen for inhibitors of the HSF1 stress pathways (Cheeseman *et al*., 2017). HSF1 is a pivotal regulator of the heat shock response under both physiological and pathological conditions (Dai and Sampson, 2016). Aberrant expression and activation of HSF1 is involved in cancer progression and drug resistance (Vydra *et al*., 2013). Bisamide (CCT251236) is orally bioavailable and successfully inhibited growth of SK‐OV‐3 human ovarian carcinoma xenografts (Cheeseman *et al*., 2017). Here, we showed that bisamide (CCT251236) dose‐dependently inhibited proliferation, migration, and invasion of PCa cells. Together our results support the potential of *PIR* as an important therapeutic target for HSPC and CRPC. Toward clinical application of bisamide, more detailed analysis of off‐target effects of si‐*PIR* or bisamide is necessary. It is important to investigate the molecular mechanism of inhibitory effects of si‐*PIR* or bisamide in detail with a current genomic approach.

## 5 Conclusions

Our results showed that expression of both strands of the *miR‐455*‐duplex (*miR‐455‐5p* and *miR‐455‐3p*) was significantly downregulated in HSPC and CRPC specimens, and thus, *miR‐455*‐duplex could act as an antitumor miRNA in PCa cells. Eight genes were regulated by *miR‐455*‐5p/‐3p, and the expression of these genes was associated with PCa pathogenesis. Among these genes, aberrant expression of *PIR* enhanced cancer aggressiveness, suggesting that *PIR* might be a promising diagnostic marker for HSPC and CRPC. Furthermore, CRPC treatment strategies targeting *PIR* may be possible in the future. Our approach based on antitumor miRNAs could contribute to the development of new diagnostic markers and therapeutic strategies for CRPC.

## Conflict of interest

The authors declare no conflict of interest.

## Author contributions

TA, SK, and NS designed the research and managed the study. YY, SS, and MK performed statistical analyses and coordinated the figures. KY, YN, and TI designed the experiments and interpreted the results.

## Supporting information


**Fig. S1.** Schematic representation of the chromosomal location of human *miR‐455*.

**Fig. S2.** Incorporation of both *miR‐455‐5p* and *miR‐455‐3p* into the RISC.

**Fig. S3.** Relationship between putative oncogenes regulated by *miR‐455‐5p/‐3p* and clinicopathological factors in PCa patients.

**Fig. S4.** Heatmaps of genes regulated by the *miR‐455*‐duplex and Kaplan–Meier patient survival curves for disease‐free survival rates.

**Fig. S5.** Direct regulation of *PIR* expression by *miR‐455‐5p* in PCa cells.

**Fig. S6.** IHC score and representative immunostaining pictures.

**Fig. S7.** Validation of si‐*PIR* function.

**Fig. S8.** Evaluation of mRNA expression of *PIR* using bisamide.

**Fig. S9.** Search for genes downstream of *PIR*.

**Fig. S10.** Kaplan–Meier curves of overall survival in other cancers due to difference in *PIR* expression based on TCGA database.

**Table S1.** Patient characteristics.

**Table S2.** Product numbers for reagents.

**Table S3.** Patient characteristics and IHC score evaluated using immunohistochemistry.

**Table S4.** Genes downregulated by si‐*PIR* in PCa cells.

**Table S5.** Genes downregulated by PIR inhibitor (bisamide) in PCa cells.

**Table S6.** Genes downstream of *PIR*.

**Table S7.** Trends of HSF1‐mediated gene expression in the presence of the PIR inhibitor bisamide.
