# Supplementary figures and images for: Pirin: a potential novel therapeutic target for castration‐resistant prostate cancer regulated by miR‐455‐5p
Source: Mol Oncol. 2018 Dec 21;13(2):322–37. doi: 10.1002/1878-0261.12405 (PMC6360383; doi:10.1002/1878-0261.12405)

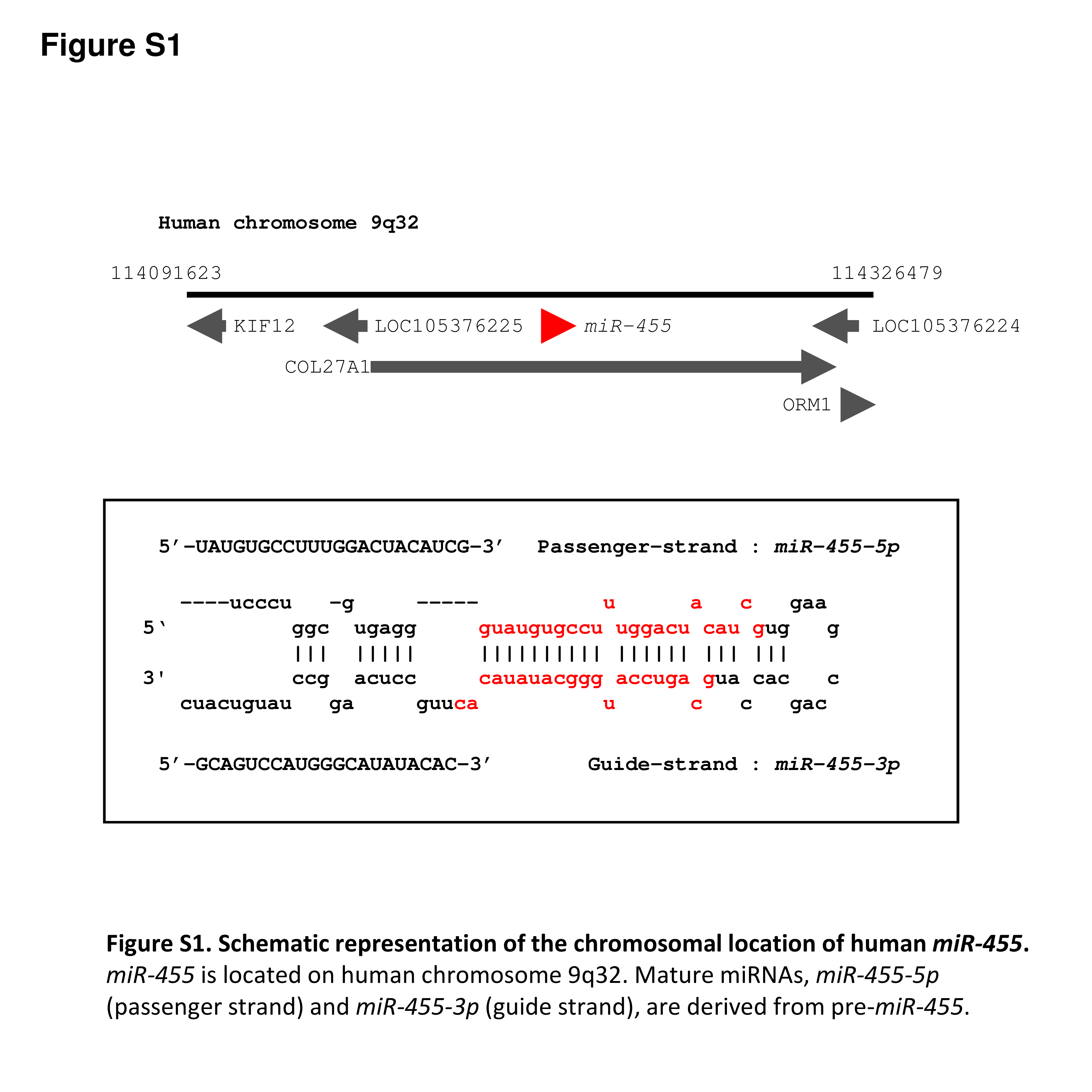

Supplement: Supplementary file 1 — Fig. S1. Schematic representation of the chromosomal location of human miR‐455. [file MOL2-13-322-s001.tiff]

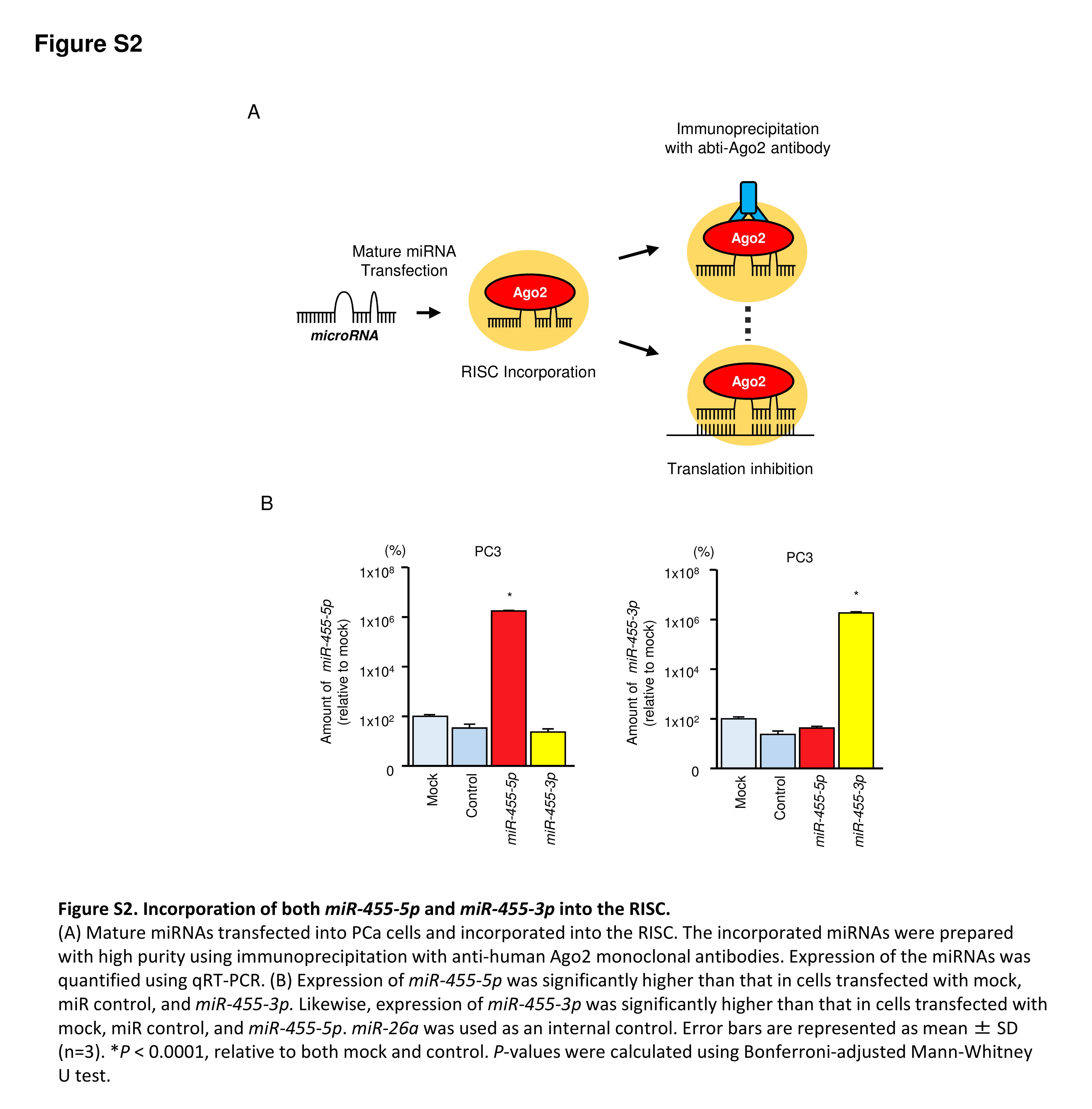

Supplement: Supplementary file 2 — Fig. S2. Incorporation of both miR‐455‐5p and miR‐455‐3p into the RISC. [file MOL2-13-322-s002.tiff]

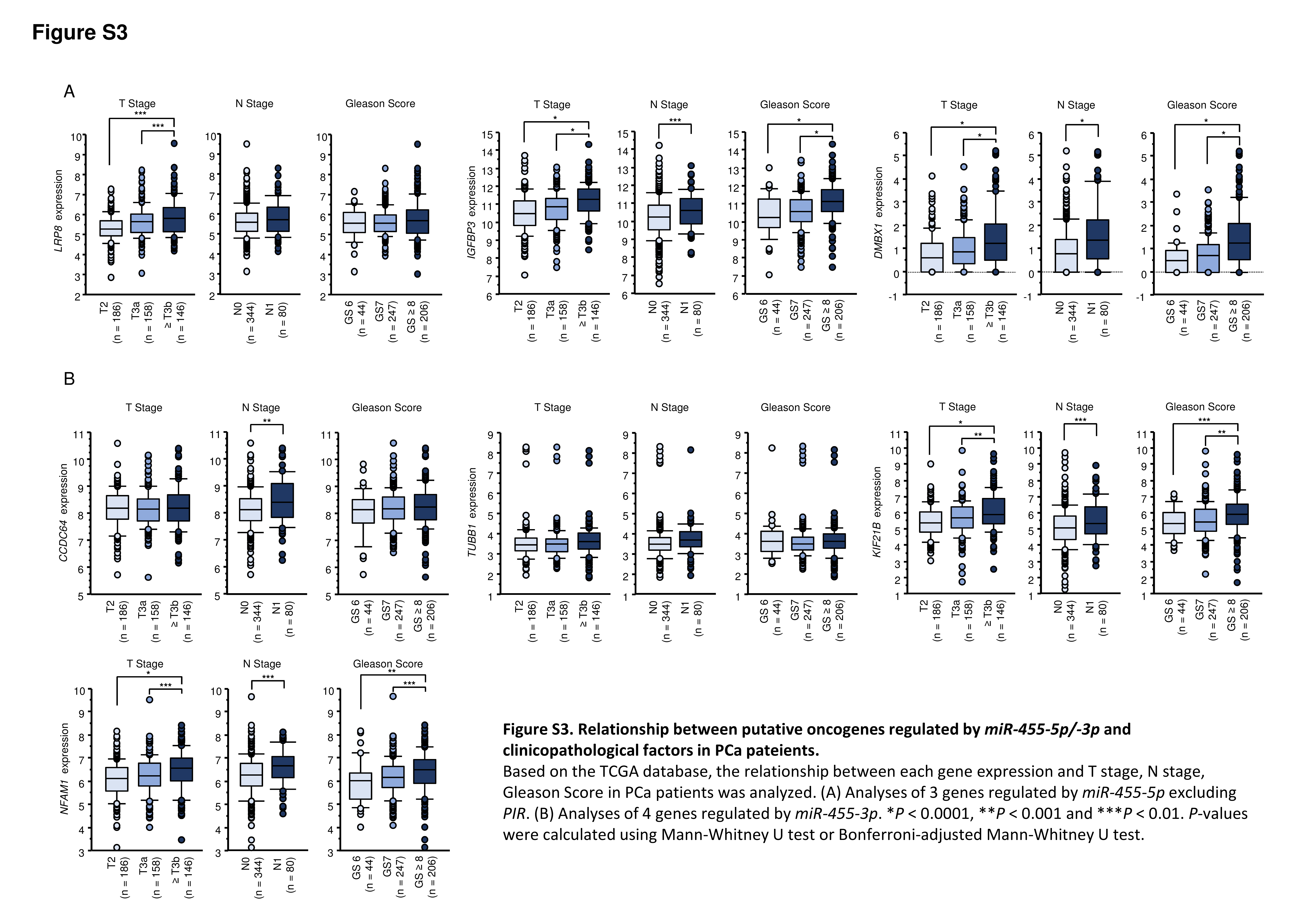

Supplement: Supplementary file 3 — Fig. S3. Relationship between putative oncogenes regulated by miR‐455‐5p/‐3p and clinicopathological factors in PCa patients. [file MOL2-13-322-s003.tiff]

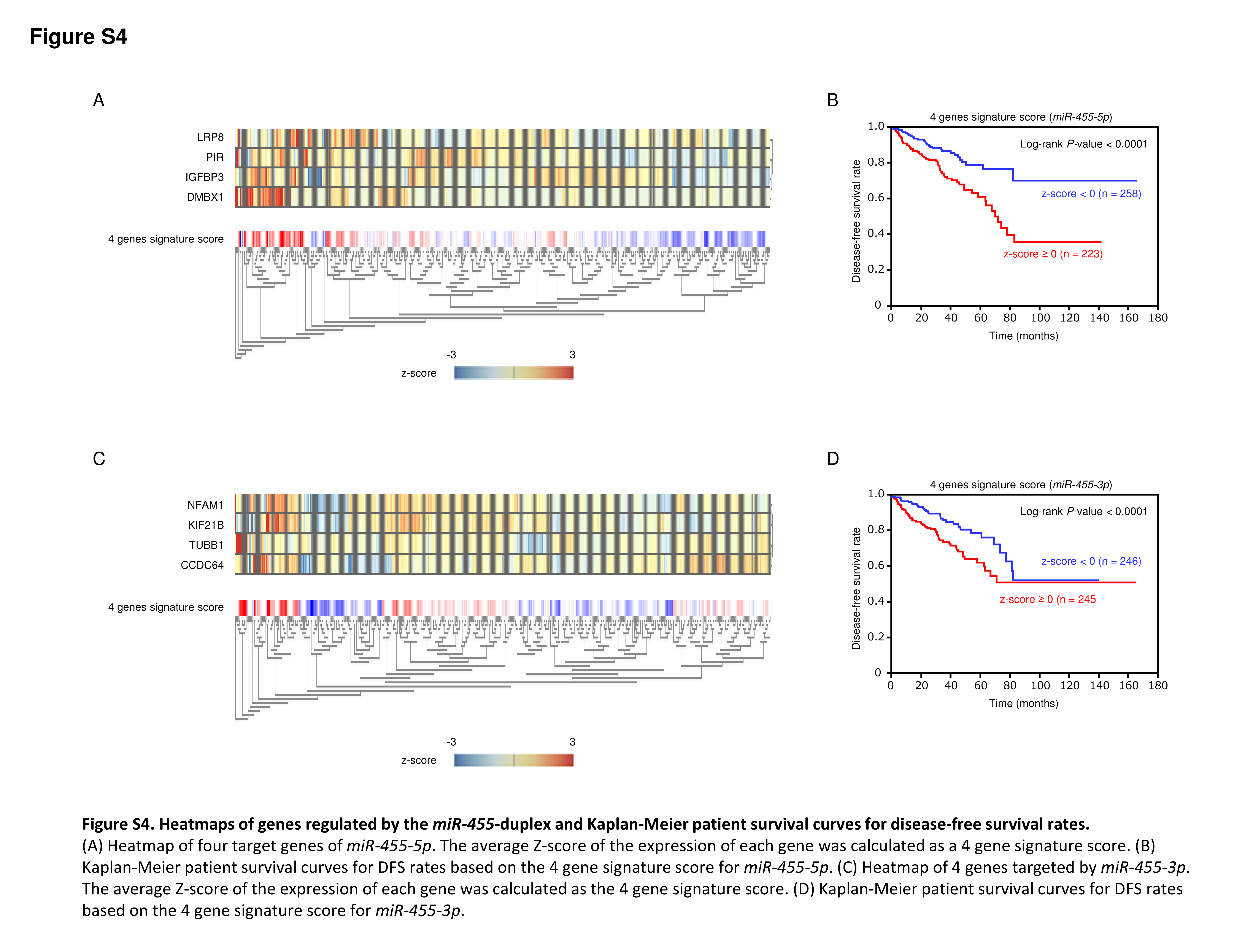

Supplement: Supplementary file 4 — Fig. S4. Heatmaps of genes regulated by the miR‐455‐duplex and Kaplan–Meier patient survival curves for disease‐free survival rates. [file MOL2-13-322-s004.tiff]

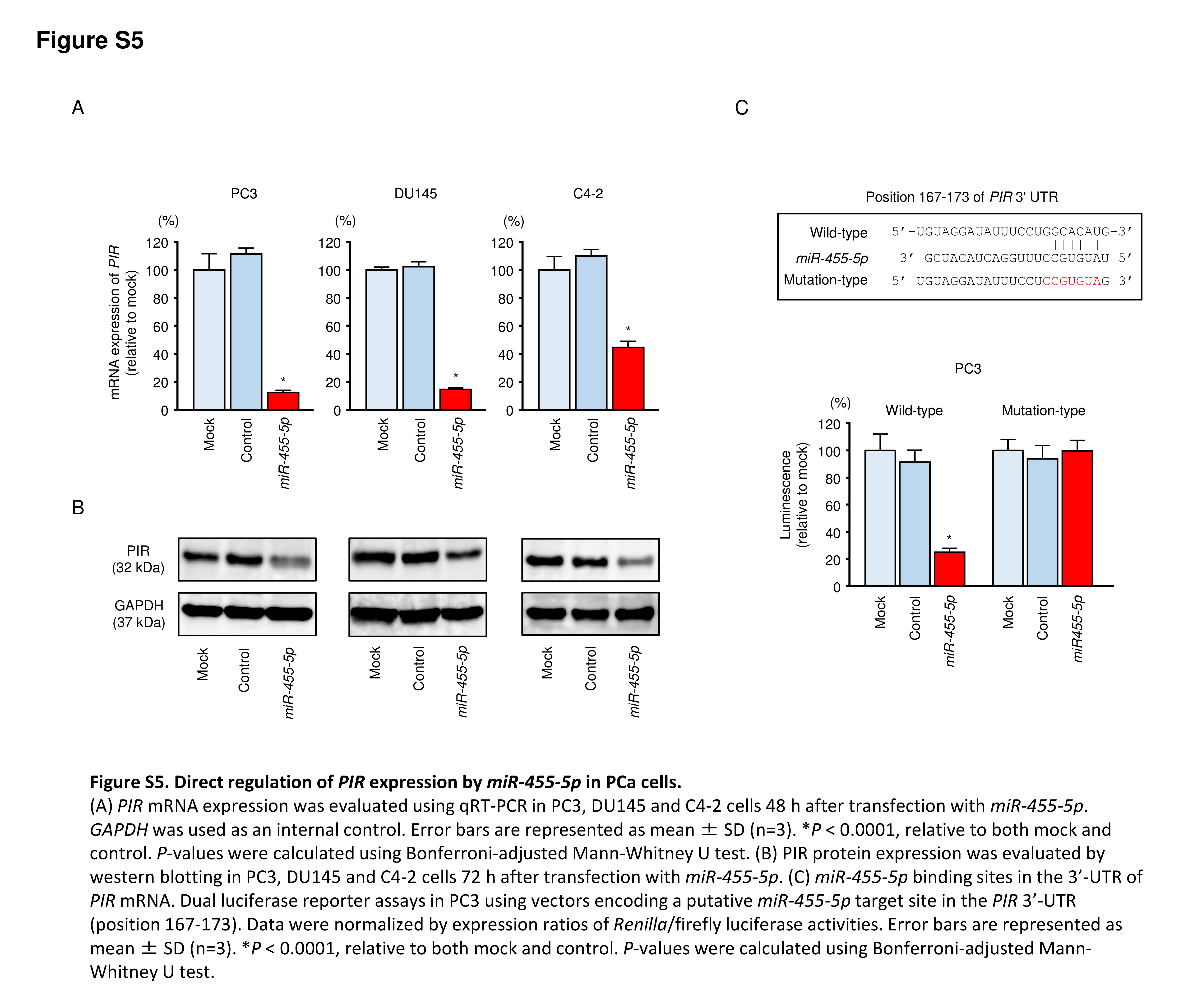

Supplement: Supplementary file 5 — Fig. S5. Direct regulation of PIR expression by miR‐455‐5p in PCa cells. [file MOL2-13-322-s005.tiff]

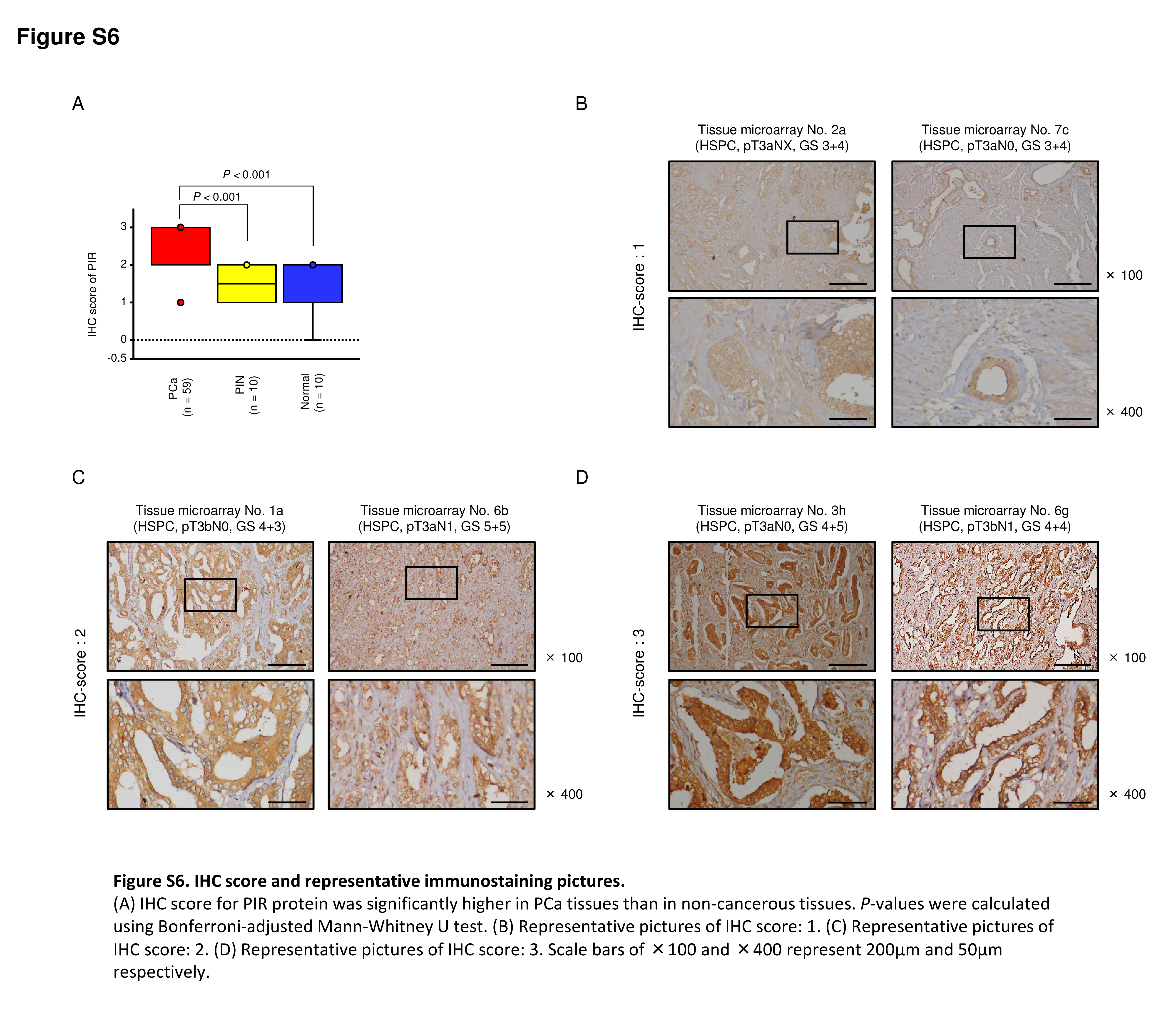

Supplement: Supplementary file 6 — Fig. S6. IHC score and representative immunostaining pictures. [file MOL2-13-322-s006.tiff]

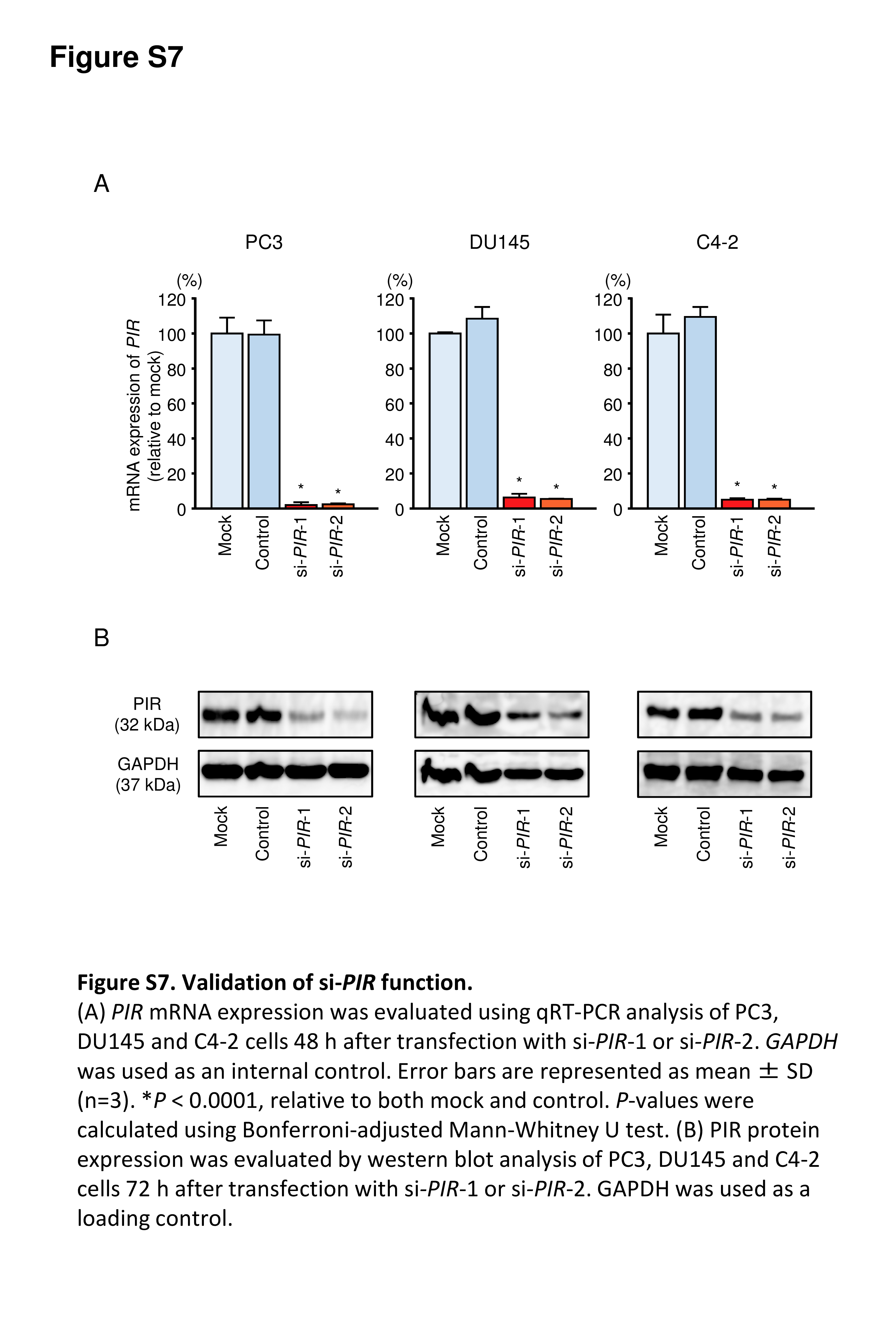

Supplement: Supplementary file 7 — Fig. S7. Validation of si‐PIR function. [file MOL2-13-322-s007.tiff]

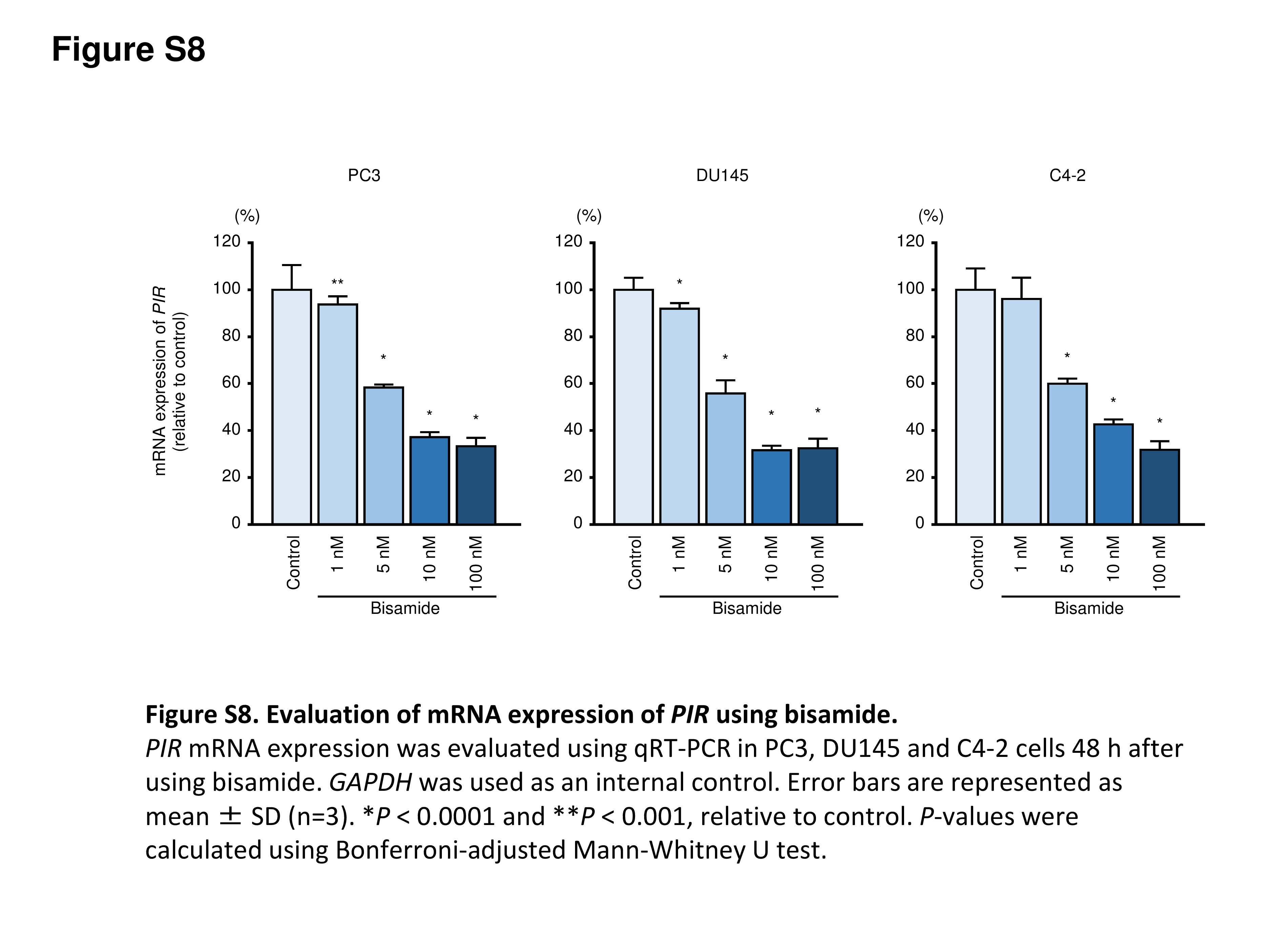

Supplement: Supplementary file 8 — Fig. S8. Evaluation of mRNA expression of PIR using bisamide. [file MOL2-13-322-s008.tiff]

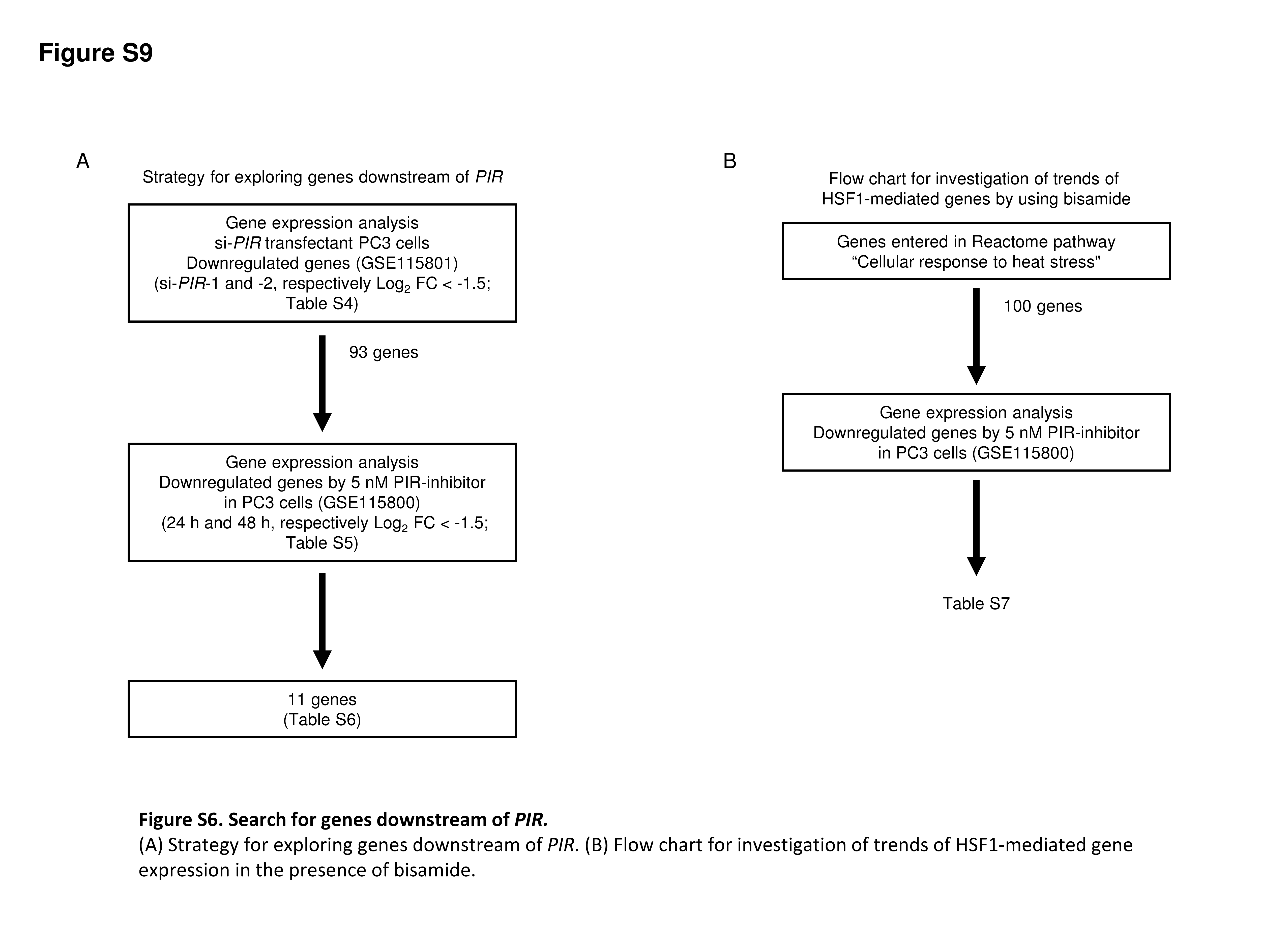

Supplement: Supplementary file 9 — Fig. S9. Search for genes downstream of PIR. [file MOL2-13-322-s009.tiff]

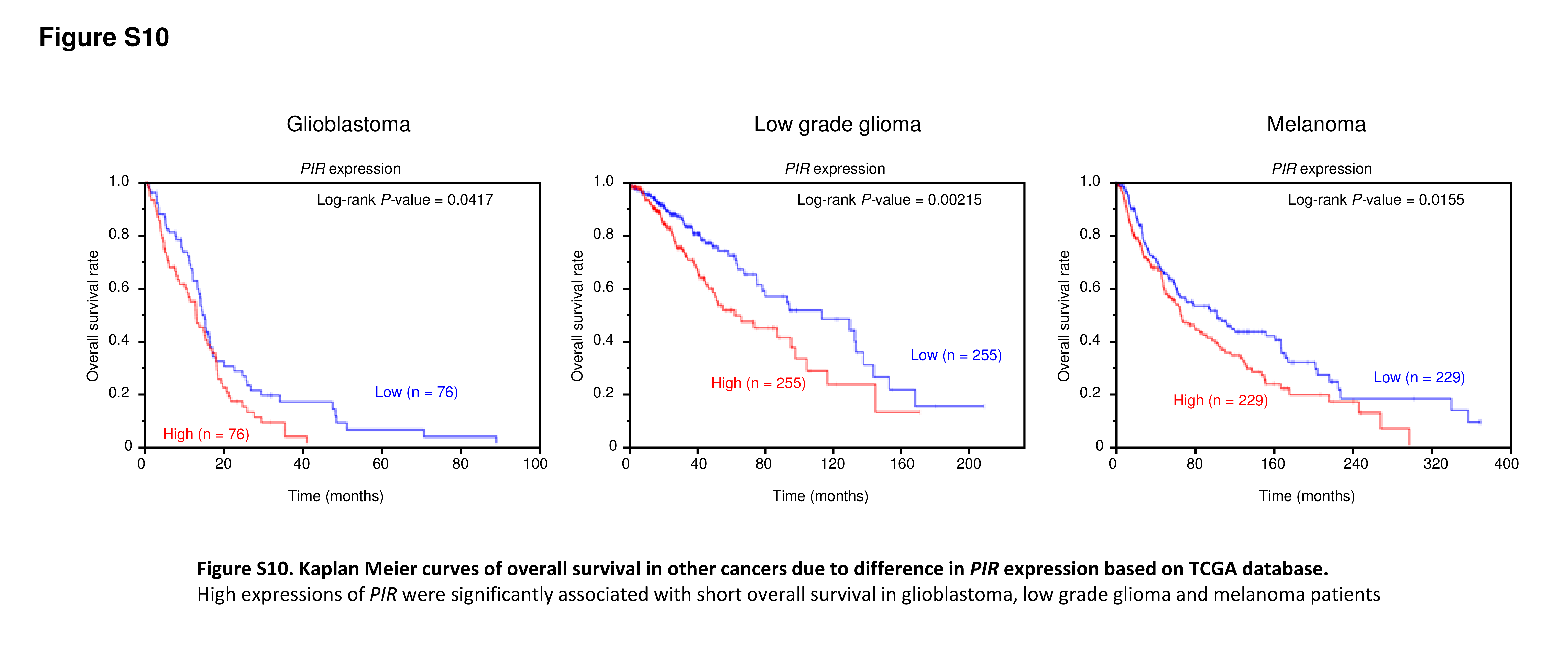

Supplement: Supplementary file 10 — Fig. S10. Kaplan–Meier curves of overall survival in other cancers due to difference in PIR expression based on TCGA database. [file MOL2-13-322-s010.tiff]
